# Serial Section Scanning Electron Microscopy (S^3^EM) on Silicon Wafers for Ultra-Structural Volume Imaging of Cells and Tissues

**DOI:** 10.1371/journal.pone.0035172

**Published:** 2012-04-16

**Authors:** Heinz Horstmann, Christoph Körber, Kurt Sätzler, Daniel Aydin, Thomas Kuner

**Affiliations:** 1 Institute of Anatomy and Cell Biology, Heidelberg University, Heidelberg, Germany; 2 Biomedical Sciences Research Institute, University of Ulster, Coleraine, Northern Ireland; 3 Department of Biophysical Chemistry, Heidelberg University, Heidelberg, Germany; Virginia Commonwealth University Medical Center, United States of America

## Abstract

High resolution, three-dimensional (3D) representations of cellular ultrastructure are essential for structure function studies in all areas of cell biology. While limited subcellular volumes have been routinely examined using serial section transmission electron microscopy (ssTEM), complete ultrastructural reconstructions of large volumes, entire cells or even tissue are difficult to achieve using ssTEM. Here, we introduce a novel approach combining serial sectioning of tissue with scanning electron microscopy (SEM) using a conductive silicon wafer as a support. Ribbons containing hundreds of 35 nm thick sections can be generated and imaged on the wafer at a lateral pixel resolution of 3.7 nm by recording the backscattered electrons with the in-lens detector of the SEM. The resulting electron micrographs are qualitatively comparable to those obtained by conventional TEM. S^3^EM images of the same region of interest in consecutive sections can be used for 3D reconstructions of large structures. We demonstrate the potential of this approach by reconstructing a 31.7 µm^3^ volume of a calyx of Held presynaptic terminal. The approach introduced here, Serial Section SEM (S^3^EM), for the first time provides the possibility to obtain 3D ultrastructure of large volumes with high resolution and to selectively and repetitively home in on structures of interest. S^3^EM accelerates process duration, is amenable to full automation and can be implemented with standard instrumentation.

## Introduction

High resolution 3D ultrastructural microscopy of large cellular volumes or entire cells is required to obtain quantitative insights into the subcellular localization, geometric arrangement and distribution of cellular components - fundamental determinants of cellular function. While the subsequent considerations in principle apply to all cell types and tissues, our focus on nervous tissue illustrates the fundamental requirements of ultrastructural volume imaging particularly well. The ultrastructural description of a single neuron can require the reconstruction of a very large volume of densely packed tissue in order to follow cellular processes such as dendrites and axons, with diameters often less than 200 nm, but nevertheless extending tens to hundreds of micrometers from the cell body. However, in neurobiology, the precise mapping of the synaptic connections of a given neuron throughout its dendritic and axonal arborisations is essential, since the position of the synapses is an important determinant of the computational properties of the neuron [1]. Moreover, the ultrastructural features of a given synapse (e.g. the number of synaptic vesicles or size of the synapse) are important determinants of the functional state of the synapses [2]. Therefore, obtaining high-resolution ultrastructural data of large tissue volumes is crucial to deepen our understanding of mammalian brain function (reviewed by [3]).

However, until now, mostly small volumes like single synaptic boutons or dendritic spines have been examined ultrastructurally in 3D by using ssTEM (e.g. [4]–[7]). Only few studies have undertaken the effort to investigate larger tissue volumes by ssTEM to obtain ultrastructural information about large structures such as elongated dendrites [8], [9] or the calyx of Held giant synapse (diameter ∼22 µm, see [10]). This lack of large volume ssTEM data is likely due to the fact that ssTEM, even when semi-automated, is time consuming [8], [11] as it took about a decade to fully image and reconstruct the entire nervous system of the small nematode *Caenorhabditis elegans* by this method [12]. Nevertheless, ssTEM has recently been adapted to reconstruct entire neurons in the primary visual cortex of mice [13].

As an alternative to ssTEM, scanning electron microscopy methods have been developed more recently that accelerate the imaging of large tissue volumes and thereby overcome a main disadvantage of ssTEM, albeit at the expense of resolution. These methods include serial block-face scanning electron microscopy (SBFSEM) [14], [15], and focal ion beam (FIB) milling [16]. Although these techniques are promising and rapidly yield high resolution data of large volumes, they require extensive equipment.

Here, we report a simple system to rapidly acquire high resolution SEM data of large tissue volumes using conventional sectioning and SEM equipment. To achieve this, we combined serial sectioning as utilized for ssTEM [17]–[19] with conventional SEM. The use of silicon wafers as a support to collect ribbons of ultra-thin sections facilitates the handling of samples and provides a direct conductive connection between the sample and the microscope stage. This conductive connection prevents charge accumulation of the sample and allows us to routinely obtain SEM images with a lateral pixel resolution of 3.7 nm×3.7 nm. Because conventional contrasting methods can be used, the image contrast equals that in TEM. At low magnification, regions of interest within a tissue section of 100 µm×300 µm (or larger) can be defined and subsequently imaged with high resolution for 3D reconstructions.

## Materials and Methods

### Fixation and tissue processing

All experiments were conducted in accordance with the German animal welfare guidelines and approved by the responsible authority (Regierungspräsidium Karlsruhe). One 16 days old Sprague Dawley rat was deeply anesthetized with isoflurane and chemically fixed by transcardial perfusion of 20 ml PBS immediately followed by 20 ml of 4% (w/v) Paraformaldehyde dissolved in PBS. The brain was removed and post-fixed over night at 4°C. 100 µm thick sections of the brain stem including the medial nucleus of the trapezoid body (MNTB) were cut on a vibratome (Sigmann Elektronik, Hüffenhardt, Germany). Sections were washed in PBS and incubated in cacodylic acid (100 mM) for 30 min. The MNTB area was excised and additionally post-fixed in 1.5% potassium ferry cyanide and 2% osmium tetroxide for 2 h at room temperature. After 3 washes in distilled water, sections were dehydrated in an ascending series of alcohol and stored in epoxy/propylenoxide (1∶1) over night. The sections were embedded in epoxy resin that was polymerized at 60°C for 36 h. All chemicals used were purchased from SERVA (Heidelberg, Germany).

### Block preparation and serial sectioning

Once the resin was firm, the block was trimmed to ∼100×300 µm using a diamond knife as described earlier [17], [18]. The block side facing the knife (bottom side) was coated with a thin layer of a mixture of Pattex glue (Henkel, Düsseldorf, Germany) and xylene (1∶ 500) to prevent sections from falling apart [18]. Long ribbons of up to 150 sections were cut at 35 nm nominal section thickness using an Ultracut S ultramicrotome (Leica, Wetzlar, Germany) equipped with a modified diamond knife angled at 35° (Diatome, Biel, Switzerland, boat custom-built). The thickness of the sections was confirmed as described previously [20]. In brief, a ribbon of 10 sections was placed on a Formvar (Plano, Wetzlar, Germany) coated slot grid that was completely embedded in epoxy resin, polymerized and cross sectioned. The thickness of the individual sections of the ribbon was then measured on a calibrated Leo EM 906 Transmission Electron microscope (Zeiss, Oberkochen, Germany). The average section thickness was 35±2 nm. Compression due to sectioning was neutralized by exposing the sample to chloroform vapour.

### Ribbon collection and contrasting

**Figure 1:**
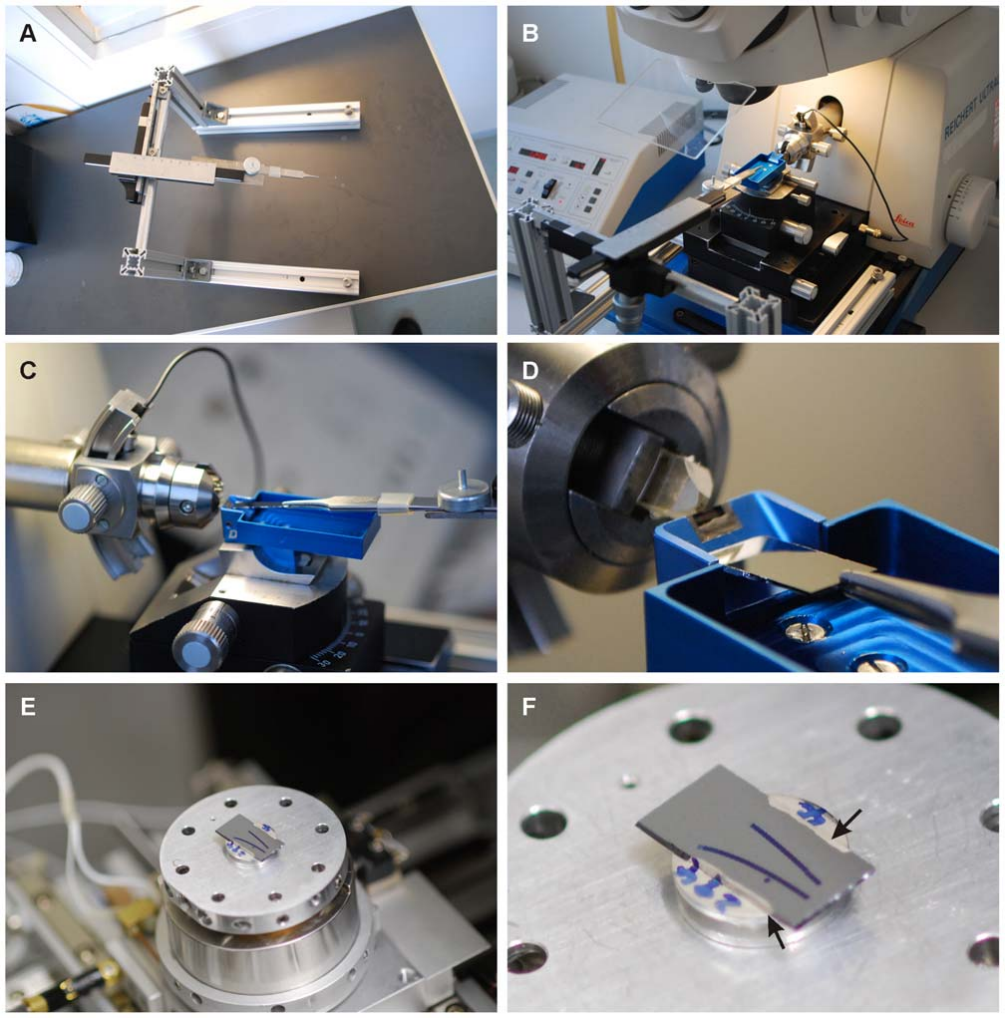
A custom-built positioning device to mount ribbons of serial sections onto a hydrophylized silicon wafer. **A:** Frame carrying an angled micromanipulator and forceps for wafer positioning. **B:** Wafer positioning frame mounted onto the ultramicrotome. C: Positioning of the wafer in the diamond knife boat (blue part). Note the extended size of the knife boat. **D:** Close-up showing the position of the wafer close to the edge of the diamond knife. A tissue block mounted on the microtome arm is visible on the left. **E:** A silicon wafer strip glued to the SEM sample holder within the sample chamber of the SEM. **F:** Close-up showing the wafer mounted on sample holder. The wafer contains two ribbons (black stripes). A jumper link was used to establish a conductive connection between the wafer and the sample holder (arrows).

Silicon wafers (type/dopant: N++/As, growth method: CZ, resistivity: <0.006 Ωcm, thickness: 525±25 µm, front surface: polished, back surface: etched, cut to 30×5 mm pieces, Si-Mat Silicon Materials, Landsberg, Germany) were cleaned and hydrophylized by incubation in sulphuric acid and 33% perhydrol (1∶1) for 45 min. After 3 washes in distilled water, the wafer was mounted via forceps (FST Student Dumont #5) onto a custom-built manual micromanipulator device attached to the ultramicrotome (Fig. 1, blue print available from the authors) that dipped the wafer into the knife boat. While cutting, the ribbon floated onto the wafer which was slowly retracted, thereby exposing the ribbon to air and allowing it to dry. Counterstaining of membranes was achieved by application of saturated uranyl acetate solution (16 min) followed by 3 washes in distilled water and incubation in lead citrate solution (8 min) in a CO_2_ free environment (modified Reynolds-procedure). Sections were again washed three times in distilled water and air dried before mounting the wafer onto a metal sample holder using conductive silver (Plano, Wetzlar, Germany) (see Fig. 1E, F).

### SEM Imaging

Scanning electron microscopy was performed using a LEO Gemini 1530 equipped with a field emission gun (Zeiss, Oberkochen, Germany). Best results were obtained using the in-lens detector at 1.8 mm working distance, 60 µm aperture and 3 keV acceleration voltage [16]. The images were inverted to achieve TEM-like representations. Images of 3072×2304 pixels were taken at 10,000 fold magnification (3.7 nm/pixel) covering 96.9 µm^2^ of tissue. The acquisition of a full frame image took ∼3 min with a dwell time of ∼40 µs/pixel.

### 3D reconstruction

A series of 100 consecutive electron micrographs of the calyx of Held synapse in the MNTB was reconstructed in 3D using OpenCAR software ([10]; available from http://opencar.ulster.ac.uk). Electron micrographs were aligned manually using prominent structures present in two adjacent images as landmarks. Subsequently, the plasma membrane, mitochondria, active zones and synaptic vesicles were manually traced in each section resulting in a set of contours representing each of these elements. The contours were 3D reconstructed using the Delaunay-method [10], giving rise to the renderings shown in the results section. Numerical readouts of the structural features present in the sections were computed using a command-line version of OpenCAR called OpenCARnEval (source as above) allowing for bulk evaluations of structural features using batch scripts. Data is shown as mean ± s.e.m.. Amira 5.3.1 software (Visage Imaging, Richmond, Australia) was used for visualization of 3D reconstructed structures.

## Results

### Specimen mounting onto hydrophylized silicon wafers

The investigation of tissue 3D ultrastructure with conventional electron microscopes requires serial sectioning of the specimen. When preparing samples for ssTEM, serial sections need to be mounted onto slot grids, a delicate, labour-intensive procedure prone to mechanical distortion of the sample, ultimately resulting in the loss of sections or the inability to generate meaningful data from them. To circumvent these problems, we established the use of hydrophylized silicon wafer strips as a direct support for the ribbon of serial sections, thereby avoiding any manipulation of the individual sections during the mounting procedure. We constructed a device (Fig. 1) that allowed us to manoeuvre the wafer strip beneath the ribbon in the knife boat of the diamond knife, thereby capturing the floating ribbon onto the wafer strip surface without forming infoldings. This procedure ensured that entire ribbons, without individual sections folded up or lost, could be firmly attached to the wafer. As sectioning progressed the wafer was slowly retracted to capture the nascent sections of the ribbon.

The mounting device consisted of a frame carrying an angled manual micromanipulator to which forceps were reversibly attached (Fig. 1A). The frame could be mounted to the ultramicrotome thereby positioning the tip of the forceps close to the knife boat (Fig. 1B). The wafer was mounted into the forceps and positioned close to the knife (Fig. 1C, D). Subsequently, the ribbon was moved out of the water allowing the sample to air dry. After heavy metal counterstaining, the wafer with one or multiple ribbons attached to it was glued to a conventional SEM sample holder (Fig. 1E, F). To prevent charge accumulation in the sample, a jumper link between the wafer and the sample holder was established using conductive silver (Fig. 1F).

### Scanning electron microscopy of serially sectioned tissue (S^3^EM)

**Figure 2:**
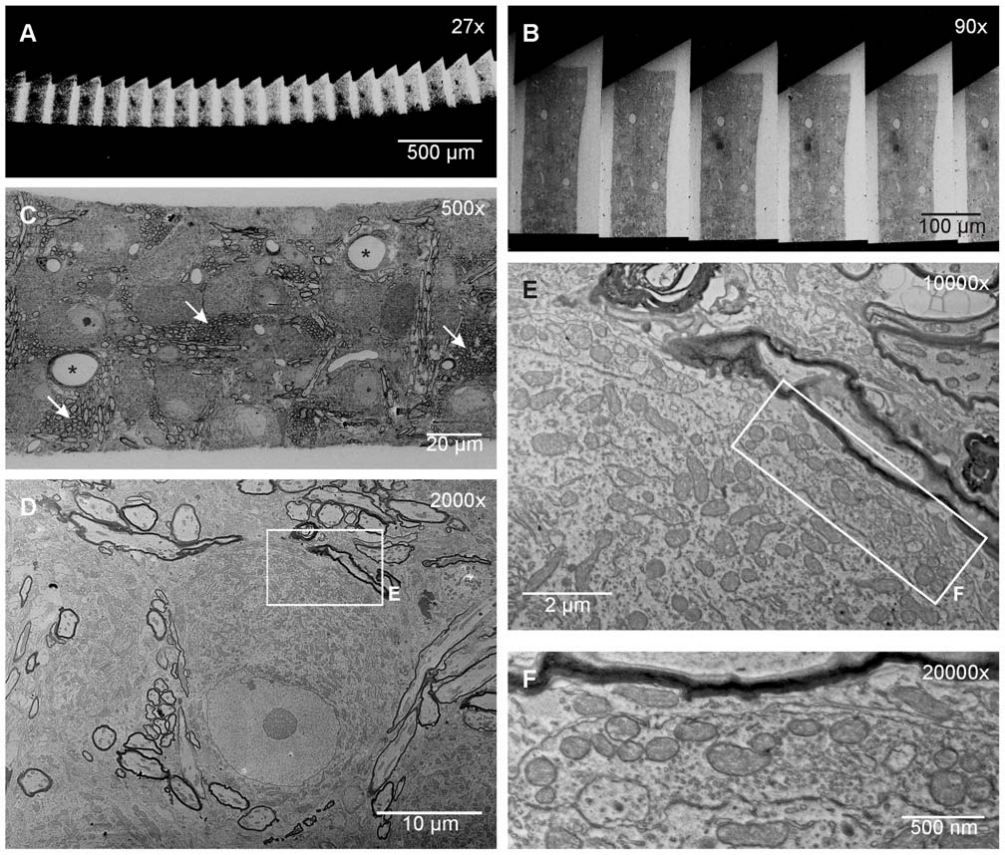
High resolution images can be obtained from ribbons of serial sections via SEM. **A:** Low magnification SEM image of a ribbon mounted on a silicon wafer. **B:** Low magnification images of sections cut in trapezoid shape to determine the orientation of the tissue. The sections are evenly space by resin throughout the ribbon. **C:** Intermediate magnification SEM images that allow the identification of landmarks in the tissue like blood vessels (asterisks) and axon bundles (arrows). **D:** Intermediate magnification overview image of a MNTB principle neuron. Boxed area is shown at high magnification in **E**. **E, F:** High magnification (10,000fold in **E**, 20,000fold in **F**) SEM images of a calyx of Held synapse segment. Boxed area in **E** is shown at higher magnification in **F**.

**Figure 3:**
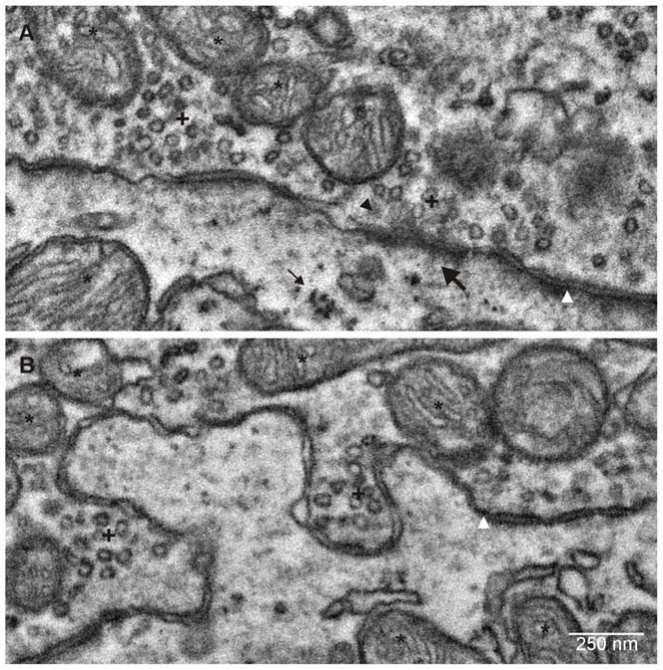
S^3^EM provides TEM-like images of cellular ultrastructure. **A, B:** SEM images of subcellular structures acquired at 10,000fold magnification (3.7 nm/pixel). Mitochondria (*), synaptic vesicle clusters (+), active zone (black arrow head), plasma membrane (white arrow head), postsynaptic density (thick arrow), ribosomes (thin arrow).

Serial sections with a thickness of 35 nm were prepared from paraformaldehyde-fixed rat brain tissue including the medial nucleus of the trapezoid body (MNTB) in the auditory brain stem. The ribbon of sections, attached to a hydrophylized wafer, was evenly spaced as evident at low magnification SEM (Fig. 2A). Since the resin block was trimmed in a way that individual sections are trapezoidal in shape, the orientation of the ribbon in the SEM can be easily determined (Fig. 2A, B). Inspection of single sections at intermediate magnification reveals, besides multiple neuronal structures for potential in depth examination, tissue landmarks like blood vessels (Fig. 2C asterisks) or axon bundles (Fig. 2C arrows) that help to re-identify the region of interest (ROI) in adjacent sections. At increased magnification, nucleus and nucleolus, multiple mitochondria and the calyx of Held terminal directly contacting the soma (see [21], [22] for review) are clearly visible (Fig. 2D). Electron micrographs of the calyx at magnifications of 10,000 or 20,000-fold, resembling 3.7 nm/pixel and 1.8 nm/pixel resolution, respectively, reveal characteristic ultrastructural features of synaptic terminals such as synaptic vesicles and active zones (Fig. 2E, F). At similar magnification, single electron micrographs acquired with S^3^EM cover areas considerably larger (∼11 µm×8.3 µm) than those covered by conventional TEM (∼2 µm×2 µm). Another advantage of S^3^EM is that ROIs for close examination can be chosen based on low magnification images of even lager areas (hundreds of µm^2^, Fig. 2C). Acquiring overview images did not result in notable decreases in the quality of high magnification images. Although higher resolutions can be achieved with S^3^EM (Fig. 2F), we usually acquired images for subsequent 3D reconstruction at 10,000-fold magnification (3.7 nm/pixel, segments from ∼30 calyces have been imaged in various projects) at which all subcellular synaptic structures are readily appreciable in a quality comparable to traditional TEM images. For example, the course of the plasma membrane, synaptic contacts including synaptic cleft, postsynaptic density and presynaptic active zone matrix can be readily identified (Fig. 3). Synaptic vesicles show typical extensions at their surface potentially reflecting synaptic vesicle proteins (e.g. [23], [24]). Mitochondria show a well preserved internal structure of membranes, outer and inner membrane can be discriminated when projection orientation allows for it. Ribosomes can be identified in the postsynaptic neuron. These observations suggest that most ultrastructural details typically present in TEM images can be reproduced with S^3^EM.

**Figure 4:**
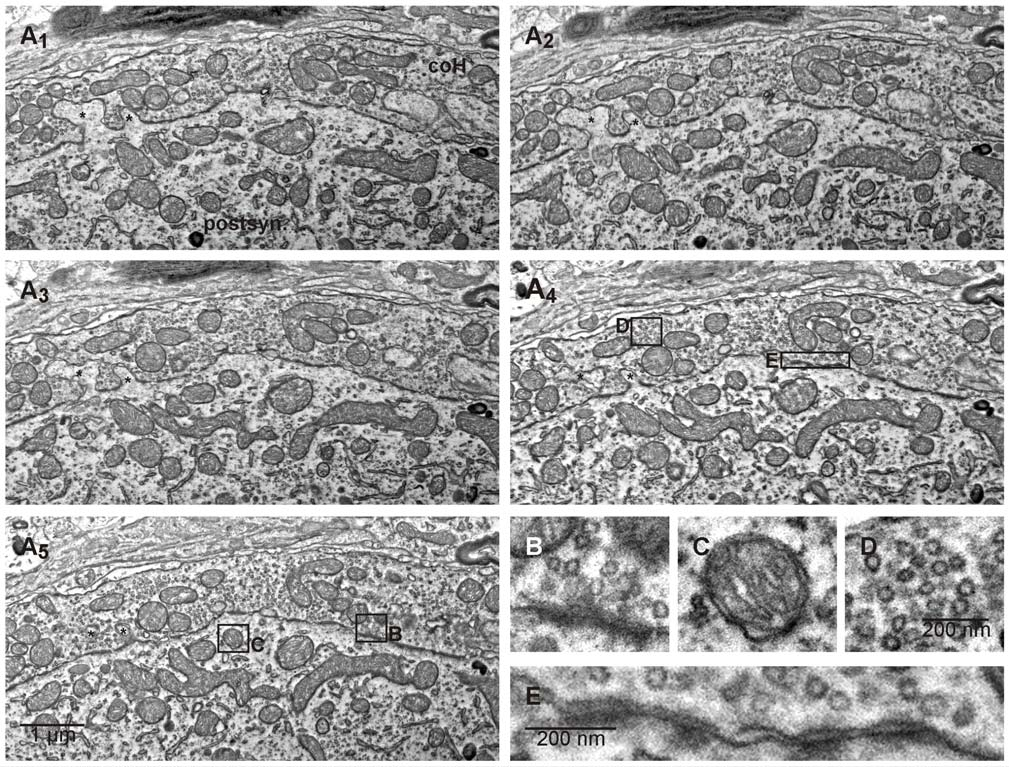
S^3^EM enables the acquisition of high resolution and high quality electron micrographs from large tissue volumes. **A:** Consecutive sections of a segment of the calyx of Held synapse (coH) and the corresponding MNTB principle cell (postsyn.) at 10,000fold magnification (3.7 nm/pixel). Cellular protrusions can be followed through the tissue (asterisks), and cellular compartments and fine structure can be recognized. Boxed areas are shown in detail at smaller scale in **B–E**. **B:** Detailed image of a synaptic release site, characterized by the cluster of synaptic vesicles and the electron-dense postsynaptic density. Scale as in D. **C:** Detailed image of a mitochondrion. The cristae are clearly visible. Scale as in D. **D:** Detailed image of a synaptic vesicle cluster. **E:** Detailed image of a part of the synaptic cleft.

Figure 4A shows a segment of a calyx of Held and its corresponding postsynaptic target cell in five consecutive sections out of series of 100 sections, imaged at 3.7 nm×3.7 nm lateral and 35 nm axial resolution (Movie S1). The sections display a number of cell biologically relevant features of this synapse, e.g. as spine-like protrusions of the postsynaptic cell that are enclosed by the presynaptic terminal (Fig. 4A asterisks, see also [10]) and can be followed through all five sections shown here. However, these protrusions are only clearly identifiable as such when followed through consecutive sections while they could be misinterpreted as cisternal structures in a single section (Fig. 4A_5_). While providing a large image area, this imaging mode operates at a high resolution as illustrated in the magnifications shown in Fig. 4B–E. Small synaptic organelles such as synaptic vesicles and fine cellular structures like mitochondrial cristae are easily detectable (shown at higher scale in Fig. 4D and C, respectively). Moreover, the conventional heavy metal counterstaining allows for the detailed investigation of membranous structures like the synaptic cleft (Fig. 4E) and presynaptic release sites which are characterized by a presynaptic vesicle cluster opposed to a thick, electron-dense structure in the postsynaptic cell (postsynaptic density) (Fig. 4B). Hence, the S^3^EM technique allows the high resolution ultrastructural description of structures in large tissue volumes with conventional sectioning and SEM equipment.

### 3D reconstruction of a calyx of Held fragment from high resolution S^3^EM images

**Figure 5:**
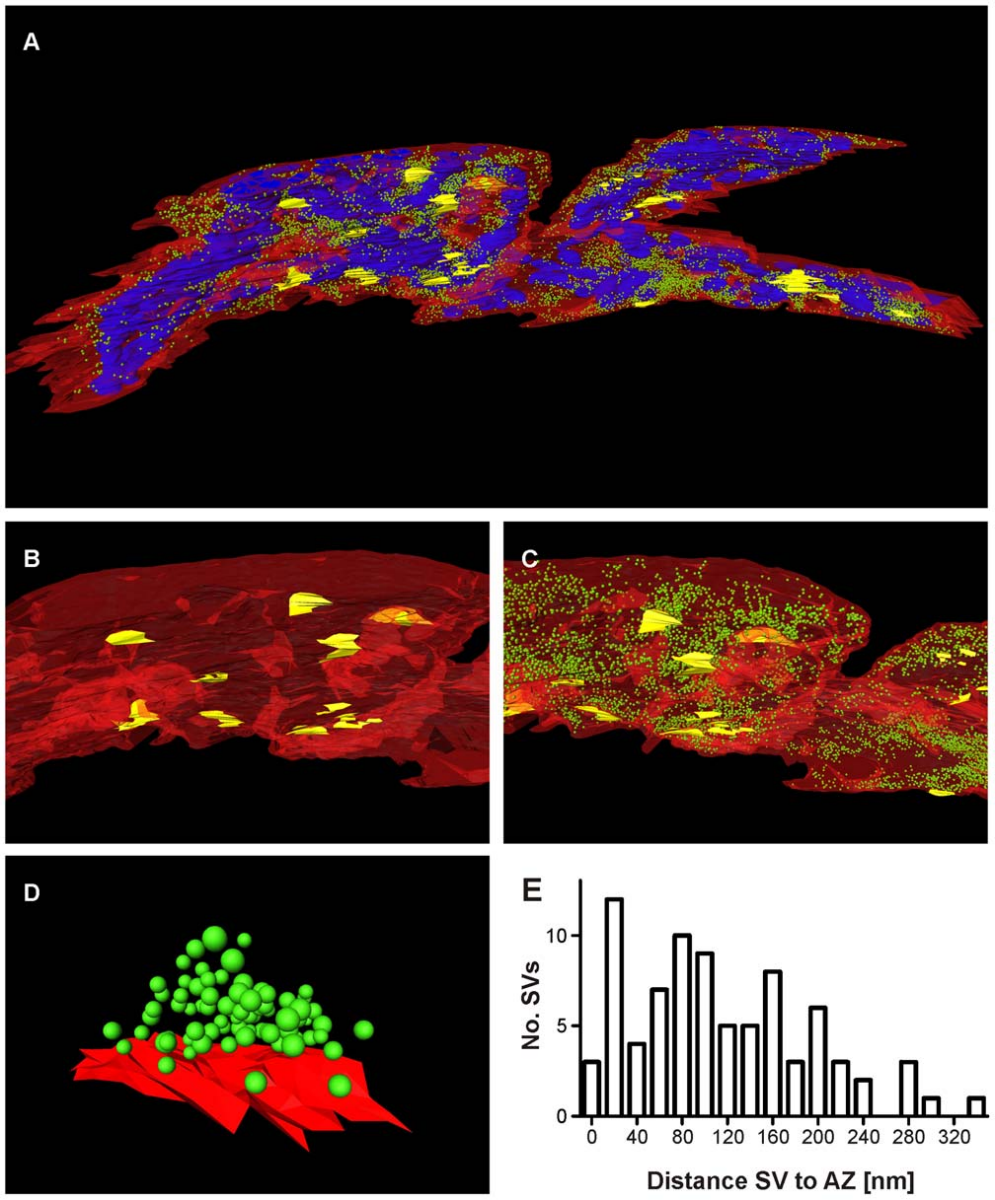
3D reconstruction of a calyx of Held segment based on SEM images obtained from 100 consecutive sections. **A:** Full reconstruction of a 31.7 µm^3^ calyx segment. The innervation side is orientated to the front. Red: plasma membrane; yellow: postsynaptic densities; blue: mitochondria; green: synaptic vesicles. **B:** Close up showing only plasma membrane and postsynaptic densities (colours as in **A**). **C:** Close up showing plasma membrane, postsynaptic densities and synaptic vesicles (colours as in **A**). **D:** 3D reconstruction of a single release site (colours as in **A**). **E:** Histogram of the distribution of distances between the presynaptic membrane and the synaptic vesicle membranes based on the release site shown in **D**.

To demonstrate that biologically meaningful results can be obtained with S^3^EM, we acquired electron micrographs from the same segment of the calyx of Held synapse in 100 consecutive sections covering 11 µm×8.3 µm×3.5 µm of tissue volume at 3.7 nm×3.7 nm lateral pixel resolution and 35 nm axial physical resolution (Movie S1). Since the ROI had to be re-defined on every single section before image acquisition, the z-alignment was not maintained and the sections had to be aligned before reconstruction (Movie S2). The aligned images were then used to manually outline the structures of interest (calyx of Held plasma membrane, mitochondria, postsynaptic densities and synaptic vesicles) using appropriate reconstruction software (OpenCAR [10] here, but *Reconstruct* [25] is also suitable). 3D reconstruction was performed based on these outlines using the Delaunay-method (Fig. 5, Movie S3). The resulting 3D representation revealed a synapse surface of 224.7 µm^2^ enclosing a synaptic volume of 31.7 µm^3^. Within this volume the geometrical features of big structures as mitochondria as well as of tiny ones like synaptic vesicles can be assessed (Fig. 5A, C). Rendering the postsynaptic densities in the plasma membrane of the postsynaptic cell allowed us to determine the position and area of synaptic release sites at which synaptic transmission takes place (Fig. 5A–C). One such release site was reconstructed independently (Fig. 5D), taking the presynaptic plasma membrane as a reference point to determine the distances between individual synaptic vesicles and the active zone plasma membrane (Fig. 5D, E). Based on these distances, we found that the release site contained three morphologically docked synaptic vesicles (membrane to membrane distance between synaptic vesicles and the release site <10 nm, see also [10]). Additionally, we extracted the diameter of the synaptic vesicles and the area of the particular release site (45.9±0.7 nm (n = 82 synaptic vesicles) and 0.168 µm^2^, respectively) from the reconstruction. All properties reported here are in good agreement with previously published ssTEM data [10], [26], thereby confirming the accuracy of the S^3^EM method. Starting with the preparation of the tissue block, the experiment presented in this paragraph including the analyses was carried out by a single person within a single week of regular working hours.

## Discussion

Here, we present S^3^EM as a tool to rapidly obtain high quality, high resolution ultrastructural data of large tissue volumes for subsequent 3D reconstruction, using conventional electron microscopy equipment.

Most studies quantitatively assessing 3D ultrastructural features of cellular and subcellular structures in the nervous system use ssTEM to obtain high resolution images for 3D reconstructions (e.g. [8], [9]). However, this method has certain disadvantages, especially when it comes to the reconstruction of large tissue volumes. The advantages of S^3^EM are already evident during sample preparation, because collecting ribbons of serial sections on hydrophylized silicon wafers avoids the error-prone, labour intensive collection of serial sections onto slot grids. Moreover, the ribbons are mounted as a whole, making it less likely to lose individual sections during the cutting procedure. The firm self-attachment of the ribbons onto the wafer prevents the folding up of parts or entire sections. Such foldings limit the examination area and hamper the acquisition of high quality data. In addition to the fact that S^3^EM accelerates and simplifies sample preparation significantly, S^3^EM has the advantage of being faster during image acquisition due the larger size of the imaged field of view in comparison to conventional TEM. Thus, fewer images have to be taken per section, thereby over-compensating the longer acquisition time per single image when using SEM. The acquisition of 100 consecutive sections used for the reconstruction presented here took about ∼7 hours (∼4 min/image, 3 minutes scanning time, 1 minute focus readjustment), the subsequent manual alignment of the images and rendering of the structures of interest another ∼40 hours. Hence, the 31.7 µm^3^ of synaptic volume could be fully reconstructed in ∼50 hours by an experienced electron microscopist, which is significantly faster than what can be achieved with ssTEM [8], [11], [19]. However, acquisition time in ssTEM could recently be accelerted by using a CCD camera array (TEMCA) and parallel imaging [13]. Nevertheless, the process of S^3^EM described here can be further accelerated by the usage of the newest generation of SEM devices operating at dwell times of ∼20 µs/pixel (Zeiss, Oberkochen, Germany, personal communication). The overall hands-on processing time can be decreased substantially by automating image acquisition, section alignment and segmentation (e.g. [11], [27]).

Besides ssTEM, a number of SEM-based methods for 3D reconstruction of large volumes have evolved in recent years, including SBFSEM and the combination of SEM with FIB milling [14], [16]. These methods provide excellent axial alignment and z-resolution (∼15 nm [15], [16]) and are capable of generating thousands of subsequent images perfectly aligned in the z-axis. With S^3^EM, the length of the ribbon is limited by the size of the knife boat (∼250 sections of 100 µm width on a 3 cm wafer) and although multiple large ribbons can be cut from the same tissue block, the microtome has to be reset which may lead to altered thickness of the first section of the new ribbon, or, more unlikely, the loss of this section. Nevertheless, SBFSEM as well as FIB milling also have some disadvantages: (1) since blocks of tissue are examined blindly, ROIs can not be chosen; (2) the tissue can not be re-examined since it is destroyed during the process; (3) the tissue needs to be stained en-block, which is difficult to achieve with larger blocks of complex tissue and (4) both methods require extensive, costly non-standard equipment. SFBSEM has the additional problem that the lateral resolution that can be routinely achieved at the moment is ∼4fold lower than with S^3^EM, ssTEM or FIB milling (16.5 nm/pixel [15] as compared to 3.7 nm/pixel (this study), 2.2 nm/pixel [8] and 4 nm/pixel [16], respectively).

Two other approaches using serial sectioning in combination with SEM have been developed recently: (1) automated tape-collecting ultramicrotome SEM (ATUM-SEM) [28], [29] and (2) array tomography [30]–[32]. ATUM-SEM uses an electron opaque tape to continuously collect serial sections into ribbons of literally infinite length. However, preliminary reports describing this instrument [28], [29] suggest that sophisticated custom-built equipment is required to establish this technique within an existing electron microscopy environment. Array tomography on the other hand is primarily a toponomic technique in which ribbons of ultra-thin sections (70–200 nm) are subjected to multiple rounds of antibody labeling [30], [31]. Subsequent to antibody labeling the LRWhite-embedded sections can be inspected by SEM after carbon coating [30], [31]. However, the relatively poor ultrastructural preservation of LRWhite-embedded tissue [33] and the less favourable conductive properties of carbon-coated glass slides that have to be used in array tomography, result in ultrastructural representations inferior to those delivered by S^3^EM.

A further method to obtain high-resolution and high-quality ultrastructural data is electron tomography. However, the maximal section thickness is, depending on the acceleration voltage, approximately 1 µm. This makes electron tomography a powerful tool for the investigation of rather small volumes like single hippocampal synapses (e.g. [34]–[36]) but limits its usefulness for the investigation of large volumes. Although it is possible to obtain high-resolution 3D reconstructions based on tomograms of consecutive sections [37], the 3D assembly of the individual tomograms is demanding. Moreover, this approach requires extremely expensive high-voltage EM tomographs, not affordable for many laboratories.

In summary, the S^3^EM method introduced here provides a powerful tool to achieve high-resolution, high-quality ultrastructural data of large volumes in a relatively short time by combining conventional serial sectioning and SEM. S^3^EM is faster and less error-prone than ssTEM while yielding comparable resolution. It is less expensive than SBFSEM, FIB milling, TEMCA and electron tomography, because it can be performed with conventional electron microscopy equipment. Additionally, S^3^EM has the potential to be automated with regard to image acquisition, section alignment and image segmentation similar to what has been developed for ssTEM ([11] and references therein), thereby further accelerating data generation and analysis.

## Supporting Information

Movie S1 **Image stack consisting of 100 consecutive sections (raw data as recorded with the SEM at constant acquisition settings) covering 11 µm×8.3 µm×3.5 µm of tissue volume at 3.7 nm×3.7 nm lateral pixel resolution and 35 nm axial physical resolution.** Note that the sections are not aligned, hence jitter reflects the slice-to-slice variability in manually identifying the region of interest. The calyx diagonally spans the image from the upper left to the lower right corner. The principal cell occupies the lower left side and an axon appears on the upper right side of the image.
(AVI)

Movie S2 **Image stack consisting of 40 cropped, aligned and greyscale-adjusted S^3^EM images showing a portion of the calyx terminal (taken from the upper left corner of Movie S1).**
(AVI)

Movie S3 **3D representation of a calyx of Held segment reconstructed from 100 consecutive SEM sections.** Red: plasma membrane; yellow: postsynaptic densities; blue: mitochondria; green: synaptic vesicles.
(AVI)
